# Editorial: Coupling in biological systems: Definitions, mechanisms, and implications

**DOI:** 10.3389/fnetp.2022.1076702

**Published:** 2022-12-13

**Authors:** Christoph Schmal, Sungho Hong, Isao T. Tokuda, Jihwan Myung

**Affiliations:** ^1^ Institute for Theoretical Biology, Humboldt University, Berlin, Germany; ^2^ Computational Neuroscience Unit, Okinawa Institute of Science and Technology, Okinawa, Japan; ^3^ Department of Mechanical Engineering, Ritsumeikan University, Shiga, Japan; ^4^ Graduate Institute of Mind, Brain and Consciousness (GIMBC), Taipei Medical University, Taipei, Taiwan; ^5^ Brain and Consciousness Research Centre (BCRC), TMU-Shuang Ho Hospital, New Taipei City, Taiwan

**Keywords:** circadian clock, coupling, biological network, entrainment, zeitgeber, suprachiasmatic nucleus (SCN)

Biological systems exhibit an enormous complexity. Their temporal evolution ubiquitously depends on non-linear interactions between non-identical, heterogeneous entities such as molecules, cells, tissues, organs, or organisms. As a result, the observed dynamics at the ensemble level show emergent phenomena that can only occur at the macroscopic scale and cannot be understood solely from the intrinsic behavior of its individual constituents, limiting the success of traditional reductionist approaches (Wolkenhauer and Green, 2013). The entities involved can exist in different positions of space and time and may span across various scales.

The process of interaction or information exchange between two or more entities in a given physical, biological or chemical system is often referred to as “coupling”. Using mathematical parlance, the dynamical evolution of a given part in a *coupled* system depends on the present or former state of other parts in the overall system.

**FIGURE 1:**
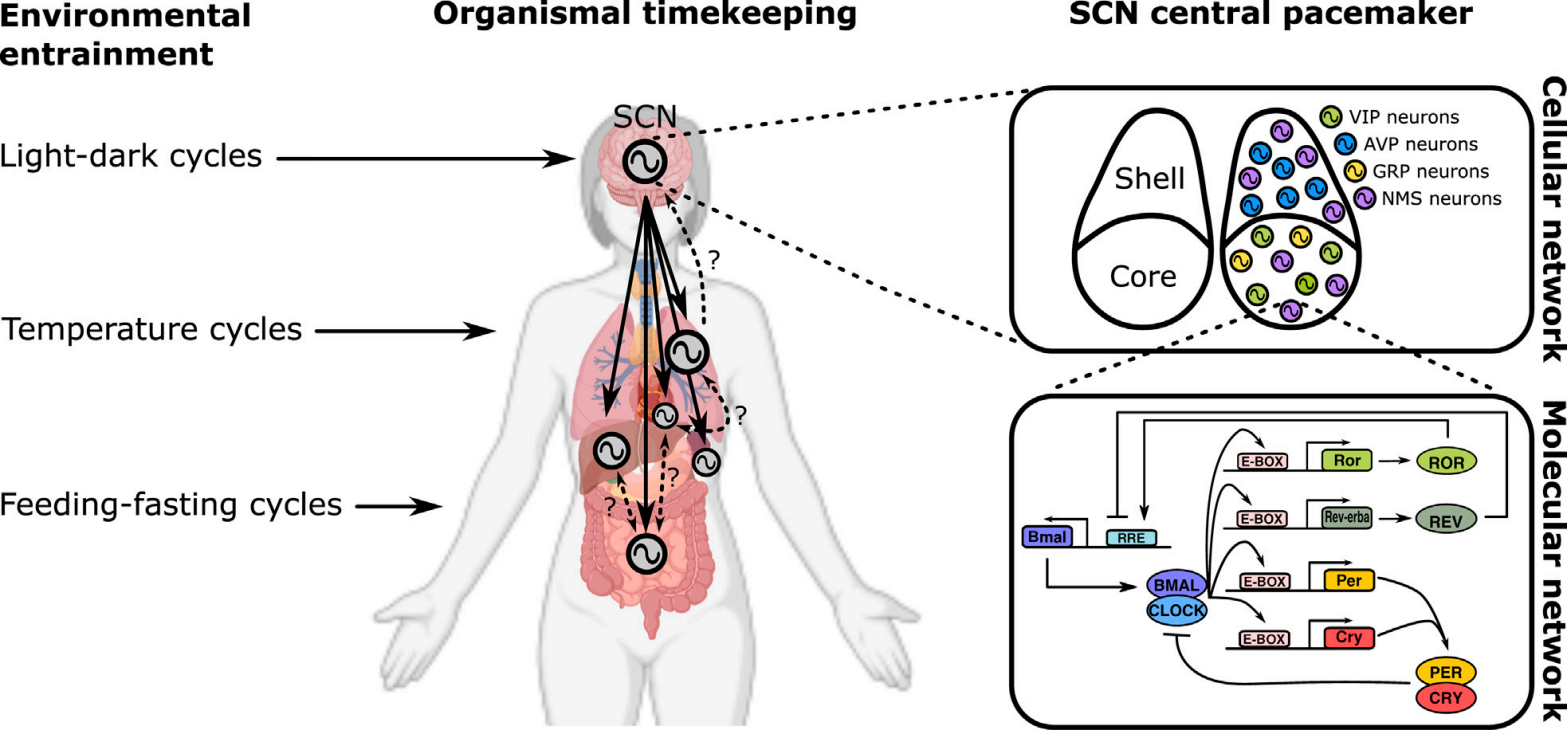
Multiscale organization of circadian clock networks. Circadian clocks exist in all scales of biology, and so does the coupling among them. The scale spans from the whole organism (far left) to molecules within a single cell (bottom right).

In multicellular systems of neurons, the emergent dynamics is the neural network computation (Figure 1 *upper right*), governed by schemes of synaptic coupling (i.e., connectome). Likewise, the macroscopic output of physiology is a result of multi-organ coupling throughout the body (Figure 1 *middle*). At the organismic level, coupling between the environment and the body enables adaptation (Figure 1 *left*). On the lowest end of the biological hierarchy, coupling among molecular components creates feedback networks within a single cell (Figure 1 *bottom right*).

In this Research Topic, we are particularly interested in coupled biological oscillators, i.e., systems composed of constituents that show rhythmically recurring patterns. Circadian oscillation is a ubiquitous biological phenomenon well suited for studying coupling across spatiotemporal scales. These self-sustained rhythms, with a period of approximately 1 day, are maintained in neuronal and non-neuronal systems at both cellular and organ levels.

An organism’s circadian rhythm must synchronize with the daily cycle most strongly defined by ambient light (called Zeitgeber, or “time-giver”). This uni-directional coupling between a Zeitgeber and a forced pacemaker is the simplest form of coupling, yet it can yield an astonishing variety of dynamical phenomena (Heltberg et al., 2021), in particular in response to seasonal variations of day length and luminosity (Schmal et al., 2020; Burt et al., 2021). Healy et al. extend the concept of Zeitgeber to include non-photic cues such as sleep, feeding-fasting cycles, or physical activity. A host of molecular pathways are suggested, which can independently and/or synergistically entrain the clock through different molecular components such as PER1/2, CRY1/2, BMAL1/CLOCK, and DBP. Grabe et al. mathematically explore this in a two-oscillator system simultaneously entrained by photic and non-photic Zeitgebers. The molecular circadian clock network is composed of two main loops of transcriptional-translational feedback: One centers on transcriptional regulation of E-box (controls, notably, *Per/Cry*) and the other on RRE (drives transcription of *Bmal1*) elements. Mammals have a central circadian clock that resides in the hypothalamic suprachiasmatic nucleus (SCN), which receives light signals directly from the retina. Using an evolutionary game theoretic framework, Spencer et al. seek for coupling topologies of the SCN that sustain circadian synchronization at minimal “cost”. The evolutionary mechanism can drive the SCN network to adopt sparse coupling against the metabolically expensive all-to-all coupling. Gu et al. consider the heterogeneous structure of the SCN network that can be divided into a dorsomedial (DM; shell) and a ventrolateral (VL; core) subregion. The authors suggest the possibility that the sparse network of the SCN can attain synchronization through small-world/scale-free coupling. The brain contains several circadian clock loci outside the SCN. Chrobok et al. discuss coupling in one stream of these clocks along the subcortical visual system (SVS). The SVS exhibits various timescales of neural firing, and circadian coupling is thought to enable multiplexing different frequency bands.

The circadian system provides an ideal context to study coupling in biological systems, which maintain oscillations in various scales of space and time. Although coupling is often a conceptualization of indirect interactions that simplifies a series of underlying processes, its consequence can be directly explored through mathematical modeling to produce experimentally testable predictions. Mathematical modeling can also provide a platform to classify the strength, directionality, and polarity of coupling in a wide range of spatiotemporal dynamics of biology while being neutral to exact details.
